# User perspectives on an electronic decision-support tool performing comprehensive medication reviews - a focus group study with physicians and nurses

**DOI:** 10.1186/s12911-016-0245-z

**Published:** 2016-01-22

**Authors:** Tuomas Koskela, Saana Sandström, Joonas Mäkinen, Helena Liira

**Affiliations:** 1 University of Tampere, Department of General Practice, Lääkärinkatu 1, 33014 Tampereen yliopisto, Finland; 2 Duodecim Medical Publications Ltd, PO Box 874, 00101 Helsinki, Finland; 3 Nordic Healthcare Group Ltd (at the time of the study), Vattuniemenranta 2, 00210 Helsinki, Finland; 4 School of Primary, Aboriginal and Rural Health Care, University of Western Australia (M706), 35 Stirling Highway, Crawley, WA 6009 Australia

**Keywords:** Computerized clinical decision support systems, Evidence-based medicine, Perceptions, Qualitative

## Abstract

**Background:**

Although a number of studies have evaluated the effectiveness of computerized decision-support systems (CDSS), there is lack of data on user perspectives, barriers, and facilitators to the implementation of CDSSs in real-life surroundings. The aim of this study was to assess individually perceived barriers, facilitators and ideas influencing the CDSS implementation and usability.

**Methods:**

In this qualitative study, five focus groups were carried out with physicians and nurses separately at the Tampere City Health Center, Finland. The participants were end-users of the EBMeDS computerized decision support system. An explorative data content analysis was applied.

**Results:**

The most important barrier to benefitting from CDSS was the lack of structured and coded diagnosis documentation and outdated medication information in the electronic health records. This led to false alerts and distrust towards the system. Among the major facilitators found were e.g. the beneficial reminders that helped practitioners take into account matters otherwise ignored; automatic glomerular filtration rate (GFR) calculations; medication safety checks; and the summaries in the single medication review at a glance.

**Conclusions:**

Physicians’ and nurses’ are keen to use the CDSS and it may enhance their inter-professional collaboration. Documenting patient information in a structured, uniform and easy manner is the essential starting point for electronic decision support. When implementing CDSS, managers need to focus on common practices in documenting structured data in their organizations in order to prevent undermining trust in the system.

## Background

Computerized decision support systems (CDSSs) provide clinicians, staff and patients person-specific, actionable recommendations or management options that are intelligently filtered or presented at appropriate times to enhance health and health care [1]. A systematic review proved that reminders are more effective than feedback in modifying physician behavior related to medication management [2]. Systematic reviews have also demonstrated that the use of CDSSs for prescribing can reduce toxic drug levels and time to therapeutic control [3–5], increase the adherence of clinicians to guideline- or protocol-based care [6, 7], decrease the rate of medication errors [7–9], enhance preventive health care delivery [7], and, ultimately, they may improve health care process measures across diverse settings [10].

EBMeDS (Evidence-Based Medicine electronic Decision Support) is a CDSS created by Duodecim Medical Publications in Finland ([11], Table 1). It has been integrated into over a dozen different electronic health record (EHR) or similar health information systems in Finland, Italy, Belgium, and other countries. EBMeDS analyzes only structured and coded patient data and provides patient-specific clinical recommendations or warnings in the form of reminders and links to guidelines. The feedback is delivered automatically to the end users at the point-of-care within the EHR user interface. The EBMeDS is evidence-based and all clinical recommendations go through an editorial process. The EBMeDS tool is available for physicians and nurses in primary and secondary care. There are different versions of the reminders focused for nurses and physicians [11]. Comprehensive Medication Review (CMR), which has been piloted in this study, is a component of EBMeDS where all available guidance for an individual patient on drug therapy is dynamically collected on demand on a single web page. As EBMeDS the CMR similarly utilizes only structured and coded patient data. In a Belgian study the majority of physician respondents were in general positive towards the ease of use and the usefulness of the EBMeDS [12].

**Table 1:** EBMeDS characteristics

Evidence-Based	The development of the decision support rules is strictly based on clinical evidence with the exception of a limited number of localized rules. The content development process has received NHS Nice accreditation in September 2011.
Context-sensitive clinical reminders and alerts	Clinical reminders based on patient data are given in real-time and automatically inside the EHR. Categories include: – Generic reminders focused on different specialties – Medication checks o interactions o adverse effects o dosing and restrictions in renal malfunction o dosing and restrictions during pregnancy and lactation o dosage and double medication o treatment suggestions (indications) – Links to guidelines
Quality measures	Quality measures describe evidence and guideline compliance in accordance with the decision support rules.
Smart forms	The patient data sent to the decision support engine is processed and populated to different electronic forms, referrals a nd calculators that physicians need in their daily work. The Comprehensive Medication Review (CMR) uses this method. The CMR could be performed for individual patients on demand.

Although a number of studies have evaluated the effectiveness of CDSSs, there is a gap of evidence on user perspectives as well as on the barriers and facilitators to the implementation of CDSSs in routine clinical practice [12–21]. In addition, little is known regarding nurses’ usage of clinical decision support systems [19]. Murphy et al. reported that clinicians received 56 alerts per day and spent 49 min processing them, making the alerts a substantial component of the daily care workflow [20]. As the consequence, this can lead to alert fatigue. CDSSs are not always utilized even if they are available [13]. In a systematic review Moxey et al. found that if a CDSS was readily available within a hospital, clinicians often failed to adopt its recommendations, ignoring up to 96 % of its alerts [14]. Nanji et al. found that about half of the CDSS alerts were overridden by the providers at the time of prescribing and about half of the overrides were classified as appropriate [21]. It is important to pay attention to the factors influencing the use of these tools in routine clinical practice.

The aim of this study was to assess the perceived barriers, facilitators and ideas influencing the CDSS usability and implementation. Moreover, we aimed to research CDSS’s influence on inter-professional collaboration within a health care unit.

## Methods

Focus groups were used to explore physicians’ and nurses’ perceptions of the CDSS for comprehensive medication reviews. The interactive discussions of focus groups generate valuable details about complex experiences and elucidate the reasons behind actions, beliefs, attitudes and perceptions [22, 23]. The participants were recruited from the Tampere Health Center (city of 220 000 population situated in Finland). Tampere Health Center is a public, municipal health center providing consultations of doctors, nurses and other health professionals, health counselling and home care services for primary care. In the Tampere Health Center the EBMeDS CDSS tool was integrated into the EHR in 2013 and the point-of-care guidelines and online reminders have been visible to physicians since then. The CMR was launched as part of EBMeDS at the Tampere Health Center in January 2014. EBMeDS and the CMR were available to nurses at the end of January 2014. None of the nurses had experiences of a CDSS prior to 2014.

A total of 9 physicians and 12 nurses participated in the study. The participants were sampled by purposive sampling, using typical case sampling method. All the physicians working in general practices had multimorbid elderly patients on their patient list, and 5 out of 9 also worked at least once a week for a couple of hours in a home care unit for elderly people. One physician worked full-time in home care in a primary care setting. All the nurses worked in a home care unit taking care of elderly patients, with each nurse caring for 60–80 patients. The nurses do not prescribe drugs, but they distribute the drugs, monitor the patients and follow-up their medication and laboratory values. The physicians and the nurses worked as pairs in home care and met face to face usually only once a week. The characteristics of the physicians and the nurses are presented in Table 2.

**Table 2:** Characteristics of the physicians and nurses interviewed in focus groups

Category	Variables	(n)
Physicians		
Gender	Female	5
	Male	4
Age	30–39	4
	40–59	3
	≥ 60	2
Education	Specialist trainee	4
	Specialist in General Practice	5
CMRs^a^ completed	1–2	1
	3–19	6
	≥ 20	2
Nurses		
Gender	Female	12
	Male	0
Age	30–39	8
	40–59	3
	≥ 60	1
CMRs^a^ completed	1–2	1
	3–19	8
	≥ 20	3

^a^CMR is a comprehensive medication review, which is an integral part of a clinical decision support system (EBMeDS®). The number of completed CMRs at the time of focus group

All the participants were trained to use the different elements of EBMeDS (focusing on the CMR) in a half-hour group training session at their workplace. The participants were encouraged and reminded by email to observe CDSS and use CMR during the following months. In addition, the EBMeDS allows to give direct feedback through feedback links next to each reminder.

Focus groups were carried out 2–3 months later in April 2014 at their workplaces. At that time some elements of EBMeDS had been visible for the physicians for a year and for the nurses for 2–3 months. Two physicians and one nurse dropped out from the study after the training session and did not take part in the focus group interview.

Three nurse focus groups (four nurses in each) were organized and two focus groups for physicians (five and four physicians in each). A semi-structured guide was followed in each interview [Table 3]. Each interview was conducted by two individuals, a moderator (TK) and his assistant (HL or JM). The interview lasted from 50 to 70 min. All the interviews were audio recorded.

**Table 3:** Semi-structured interview questions

1. Icebreaker: Could anyone share a user experience of the CMR?
2. In what kind of clinical situation did you process the CMR? – How much time did it take? – What was the consequence of this act?
3. What are the best elements of the CMR in your opinion?
4. What kind of impact has the CMR had on your work or collaboration between nurse/doctor, patient or pharmacy? Has it changed your way of working?
5. Demonstration of different elements of the CMR (which elements have you paid attention to, which have you not)
6. Do you experience any barriers to using the CMR? What limits it?
7. What kind of ideas/suggestions do you have for the future of the CMR?
8. Is there anything else regarding the CMR we haven’t mentioned yet?

The original aim of the study was to explore user experiences with regard to the CMR, but it became apparent in the focus groups that participants had more thoughts and ideas regarding CDSSs in general, which had an impact on the final research question.

In a qualitative study, the characteristics of the researchers may influence the research and the reflection. Two researchers (TK, JM) had a background as CDSS editors, two were general practitioners (GPs) (TK, HL), and one was a pharmacist (BSc) (SS). The moderator knew 4/20 of the participants. Other researchers were not familiar with the participants.

Study permission was granted by the the chief physician of Tampere Health Center. Due to Finnish ethical principles of research in the humanities and social and behavioural sciences, there was no need to apply for permission from the hospital district’s ethics committee for this voluntary interview study [24]. Written consent was obtained from all the physicians and the nurses who participated in the interviews.

### Data analysis

An explorative data content analysis was used to extract the barriers, facilitators, influence on inter-professional collaboration in a health care unit and ideas influencing the CDSS usability and implementation from the data. The data sets of nurses and physicians were analyzed separately.

First, the semi-structured audio recorded interviews were transcribed verbatim. The transcripts were read independently by all four researchers. Thereafter we coded the significant text fragments from each interview independently. We then discussed the codes and the initial themes arising from the codes. The themes arose from the data clearly and there was mutual understanding regarding them between all four researchers. We grouped the codes into 6 preliminary themes (influence on action, positive experiences, usability, opinions/perceptions, ideas, and barriers). The information in each code group was condensed, reflected on and interpreted together by all the researchers. The aim was to find all the relevant codes and to form a picture from the data of possible facilitators and barriers that influence usability and implementation of CDSS at the individual health professional level.

## Results

**Fig. 1:**
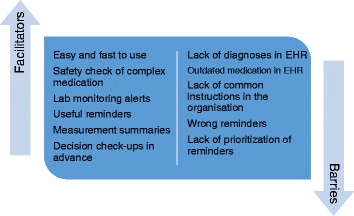
Factors influencing the use of the CDSS on health-professional level

The model including barriers, facilitators and positive ideas for beneficial CDSS use is presented in Fig. 1.

### Barriers

We found that the most significant and important barrier to benefitting from CDSS or the CMR was infrequent recording of diagnoses as coded data in the slots that have been designated to them. The structured diagnosis data was crucial for both CDSS and CMR. This was discussed at length in every interview. Further, it appeared that in many cases the medication information was not up-to-date in the EHR. This led to false alerts and reminders. Alternatively it also led to the absence of some alerts when they should have been triggered. All this undermined trust in the CDSS among physicians and nurses.

*‘The biggest problem is that diagnoses are not documented in the EHR’*

*(male GP trainee)*

The documenting of diagnoses into the EHR was unanimously experienced as complicated and time-consuming by busy practitioners. There was also discussion on whose duty it was to code the diagnoses and whether nurses should be allowed to code the diagnoses, e.g. those provided in the hospital health records but not transferred into the primary health care EHR. Nurses claimed that they were not allowed to do it and GPs argued that they did not have time to do it. Performing the CMR was also perceived to be time-consuming in cases without a previous documentation of a permanent diagnosis. The EHR also allowed a user to exit the system without documenting the diagnosis for an encounter. Moreover, it became evident that the CDSS did not take account of paused medications from the medication list in the EHR unless the drug was completely stopped by the physicians. Other barriers are presented in [Table 4].

**Table 4:** Barriers in using CDSSs as experienced by physicians and nurses

Original text fragment	Code
Physicians	
* ‘I get too many reminders for some of my patients…some of them have to be eliminated’*	too many reminders
* ‘The EHR allows me to exit the system without documenting a diagnosis for an encounter’*	lack of diagnoses
* ‘At the hospital they don’t record diagnoses in the structured way into* (our common) *EHR’*	lack of common practice in documentation in different sectors of the health care system
* ‘I have noticed that medication is not up to date in the EHR. Patients get prescriptions also from the private sector…it’s difficult to know the real medication…’*	medication not updated
* ‘the CDSS reminded me to drop the dosing of metformin (renal insufficiency) by 25 %, but I had reduced the dose already to the minimum…’*	wrong reminders
Nurses	
* ‘We are not allowed to put diagnoses in the EHR…it’s doctor’s job…’*	rules preventing effective documentation
* ‘It takes 15-20 minutes for a doctor to go through the diagnoses of one patient…who makes it and when?’*	checking up of diagnoses is time-consuming
* ‘I saw medication that was paused medication yet triggered reminders…’*	reminders launched by paused medication
* ‘Self-monitoring values were documented in the free text…’* *‘We wondered why we got a reminder to measure blood pressure…’*	lack of practice in documenting within the structured form in the EHR

### Facilitators

In general, the participants found the CDSS to be easy to use and it functioned well if the essential patient information was updated in the EHR. The facilitators are presented in [Table 5].

**Table 5:** Facilitators in using CDSS experienced by physicians and nurses

Original text fragment	Code
Physicians	
* ‘The patient has symptomatic COPD, and no information on pneumococcal vaccination was found. Consider vaccination…it’s nice that ‘someone’ has time to consider those…’* *‘It* (CDSS) *takes into account matters that I normally wouldn’t think about it…’*	beneficial reminders
* ‘I very much enjoyed getting the GFR automatically…it makes me feel useless’ (laughing)*	beneficial calculators
* ’I found this tool (CMR) to be useful when I renewed the prescriptions for patient with polypharmacy’*	the burden of adverse effect caused by drugs
* ‘As a consequence of a reminder for drug dosing in renal malfunction, I reduced the methotrexate dose, which I had forgotten’*	safety checks
Nurses	
* ‘I found the laboratory request reminders useful…’*	beneficial reminders
* ‘It’s good that you see the date of the last measurement* (in report)*…’*	measurements in a single report
* ‘It* (CDSS) *guided me to systematically check-up lab follow-up, the latest blood pressure measurements, the creatinine value and to update the medication…’*	guides to update medication and measurements
* ‘Once when my doctor was away, I used the warfarin assistant to define the dosing’*	dosing assistant

Nurses found reminders on medication monitoring useful. Also, they appreciated the condensed summary of measurements in the CMR (the most important laboratory values, the dates of last measurements and the glomerulus filtration rate (GFR), with abnormal values in red color).

*‘I found the laboratory reminders useful. When I noted the high potassium reminder I asked the GP about this’ (female nurse)*

Physicians repeatedly stated that the CDSS helped them in taking into consideration matters that they might otherwise have escaped notice. Further, they appreciated the automatic patient-specific GFR calculations and the safety checks for medication. For example, one experienced female GP received a safety alert to reduce methotrexate dosing when a decreased GFR was automatically noticed by the CDSS.

*‘As a consequence of the reminder about drug dosing in renal malfunction I reduced the methotrexate dose, which I had forgotten’.*

**Fig. 2:**
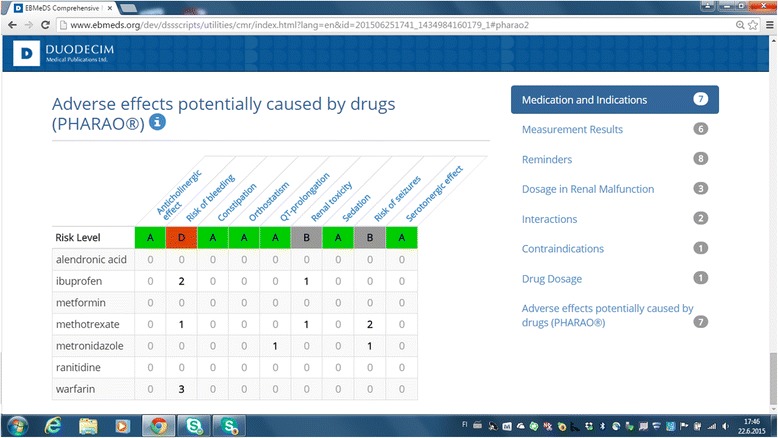
Screenshot of Pharao® element of EBMeDS® comprehensive medication review

The patient-specific multiplicative adverse effect check for polypharmacy (the Pharao® element of CMR) was also found to be beneficial. The Pharao® element evaluates the risk of the most typical adverse effects of the patient’s medication combination, such as the anticholinergic and serotonergic effects [Fig. 2].

*‘I found the adverse effect check-up tool useful when I renewed the prescriptions for a patient with polypharmacy’ (experienced male GP)*

Nurses appreciated the reminder alerts about laboratory tests, blood pressure measuring and paying attention to drug dosing in renal malfunction.

*‘Decision support guided me to check systematically for reminders about laboratory requests, blood pressure measurements. I think I started to observe more creatinine values, too. I also started to check if the patient really used all the medication he had on the list’ (female nurse)*

### Influence on inter-professional collaboration in a health care unit

The CMR was launched only two months before the focus groups were carried out. Although the physicians and the nurses pointed out that they had looked at the CMR together, they expressed that the collaboration between physicians and nurses did not change a lot as a consequence of launching this tool during the two-month period. However, nurses felt some of the CDSS alerts could be a basis for increasing collaboration with the physician.

*‘I got support from a laboratory reminder to talk about laboratory controls with our GP’*

*‘We nurses are messengers for physicians’*

One nurse suggested, for example, that the reminders and CMR could be seen as a screening tool for important medical issues to be presented to physicians. Experienced GPs in two different focus groups agreed that it could be a tool that could increase this kind of collaboration. It could motivate the creation of a common policy within a health care unit to document medication and diagnoses.

### Ideas

The shared ideas regarding the future CDSS are presented in Table 6. A GP trainee reported that it would be helpful if the reminders were prioritized with different colors and the most essential findings were placed on the top of the CMR report. Another GP trainee mentioned that it would be useful to test the potential pros and cons of the medicines with the CDSS before prescribing them.

**Table 6:** Physicians’ and nurses’ ideas of beneficial characteristics of future CDSS

Text fragment	Code
Physicians	
* ‘Could this (CDSS) screen for multipharmacy patients and recommend a comprehensive medication review for some of them?’*	screening patients for comprehensive medical review
* ‘I would like to see the most important things on the top of the medication review*	highlighting information
* Some temporary medications on the medication list, for example a short course of iron supplements, may have remained on the list beyond the intended completion date…to receive some kind of notification about this*	reminders for unnecessary medication
* ‘I would like to use this tool for getting new ideas for my patient’s medication. Just like talking with a colleague’*	getting new ideas
* ‘It could take into account the reduced dosing and GFR at the same time…’*	taking notice of the dosing of medicine
Nurses	
* ‘Helpful for me would be to see blood pressure, weight and blood sugar follow-up results at one glance’*	summary of meaningful measurements
* ' Many patients have taken antidepressants and sedatives for decades…nobody pays attention to it…I would like to see this tool reminding me to follow-up and consider discontinuation…’* (nurse talks about medication of elderly home care patients)	reminders to check-up indications for long-term medicines
* ‘We could measure pain on VAS-scale and document it in the EHR’*…(in a structured form)	utilizing pain measurements for the CDSS
* ‘I have to remind myself to measure orthostatic blood pressure from an elderly person, who is at the risk of falling…’*	Reminders to detect persons at the risk of falling
* It would be nice to have direct laboratory referrals integrated into reminders…you could click through the reminder to a lab form and set the dates for referrals…it would ease my work’*	Laboratory referrals integrated into reminders

*‘I would like to get new ideas about medication from the CDSS. Just like talking with a colleague’*

Nurses working in home care units mentioned that they would appreciate a CDSS tool that could support them in the most common clinical situations, such as with pain and constipation treatment and with preventing falls. A nurse also mentioned that the CDSS could be useful in the follow-up of the psychopharmaceutical drugs.

*‘For example, it could compare the VAS measurements for pain and pain medication…’*

## Discussion

According to our qualitative study, Finnish physicians and nurses found the CDSS and CMR useful in primary care settings if diagnoses were documented and use of medication was updated in the EHR. Based on this study the use of the CDSS is facilitated by beneficial reminders, safety checks and summaries that users find useful in clinical situations. To enable a well-functioning system, medication and diagnoses must be updated by the users. There should be common practices within organizations for documenting structured data. CDSS software should facilitate ease of documentation and produce focused and graded information at the point of care for the end-user.

In our study, the health professionals, who recently started using a CDSS system, found the lack of diagnosis codes to be the most significant barrier to using the system. Similarly, the fact that medication lists were outdated in the EHR prevented users from fully benefitting from the CDSS. This made the system incomplete and less trustworthy. Apparently, these observations turned up to be ‘control beliefs’ working against willingness to adopt the system. According to the Theory of Planned Behavior [TPB] [25, 26], such beliefs could limit the adoption of a system. In further testing of the CDSS, more effort must be made to make the system more complete with adequate with drug information data to reduce such control beliefs.

Another link to the Theory of Planned Behavior was that apparently the CDSS users were more confident of the system when it linked information to action. According to the TPB [25, 26], this is an example of a behavioural belief, which produces a favourable attitude toward the behaviour: starting to apply decision support more often in the own clinical practice.

A significant reason for the missing information related to their organization’s practices and lack of leadership regarding this. There were no common instructions to document permanent diagnoses in the study health center. Moreover, it appeared that in some units the diagnoses were documented in the free text, preventing their use in decision support.

Busy clinicians felt that updating the diagnoses in the EHR was complicated and time-consuming. This sets a challenge for the CDSS system providers since the CDSS system should be designed to support this transition.

In order to fully benefit from the potential of the CDSS and CMR systems, the healthcare documentation practices and organizational setting should be updated to exploit the utilization of the recorded patient information instead of simply recording the information. Patient information is required in an accurate structured format in the EHR system. One conclusion of our study is the need for specific management for the patient record information practices.

In our focus groups, it appeared that the lack of correct information undermined trust in the CDSS. The missing diagnoses were considered a problem. It seems that the change in practices needs support from management, e.g. instructions, as well as license for the nurses to copy the diagnoses from hospital information records to the health center EHR.

On the other hand, if diagnoses were documented and medication was up to date, the CDSS and CMR were found to be useful for both physicians and nurses in clinical work in a primary care setting. The CDSS and CMR systems have the potential to function as a tool that would enhance the culture for inter-professional collaboration in primary and home care. Both nurses and physicians found that nurses could use this as a screening tool for important medical issues. This would optimally lead to supervised medication treatment decisions by the nurses in conventional situations, while providing the physician with CDSS assistance in more complex medication situations.

### Limitations and strengths

A limitation of the current study is that participants were recruited from a single, albeit large health center; they used a single CDSS and their experience as decision support users was as yet brief. The physicians and the nurses had voluntarily participated in the focus groups. It is therefore likely that they are more interested and are more reflective about the CDSS than the average clinicians. A further important limitation is the well recognised problem that individuals do not always do what they say they do.

Two of the four researchers were CDSS developers. In the qualitative study, with subjective approach, this could have caused bias.

On the other hand a strength of the study is that it was aimed successfully at obtaining new information on the user perspective regarding the use of a CDSS system with CMR. Nurses were included in addition to physicians in the study group, facilitating a broader understanding of the different end-users of the system as well as on the system as a tool in an inter-professional collaboration in health care. In the recent review Piscotty and Kalisch pointed out that little is known how, when, and why nurses use CDSS [19]. Physician interviewees were both young and experienced [Table 2]. We also interviewed nurses and physicians in separate focus groups in order to avoid the dominance of either group in the discussion.

The data reflect the perspectives and experiences of the participants. A qualitative study in this context is important for generating hypotheses. The generalizability of the findings is limited, but this is never the intention in qualitative studies, the main aim of which is to contribute to increased understanding.

### Comparison with existing literature

Clinicians often fail to adopt CDSS recommendations [13, 14]. The results of this study emphasize some possible reasons. In the previous study of Patterson et al., the primary reason for not paying attention to clinical reminders was extensive workload. Physicians reported using clinical reminders only when they had additional time [17]. In our study clinicians also mentioned lack of time as a significant reason for not updating the diagnoses and medication. In a systematic review, users reported clinical situations in which inappropriate reminders are annoying [14]. The high frequency of reminders has been perceived as irritating in the context of a consultation and as a consequence, users have felt they may become desensitized to alerts [14, 21]. Further, the simplicity and visibility of messages were considered as key drivers of use. There have been suggestions that alerts should be graded by severity [14]. Our study underlines the same observation. Too much non-graded information on the screen could be a barrier to the beneficial use of CDSS. In addition our study highlights that reminders were perceived as inapplicable due to missing diagnostic codes or outdated medication. False alarms create a lack of trust in the CDSS and according to TPB this may lead to behavior which is a function of attitudes towards the behavior in question [25, 26]

## Conclusions

Both nurses and physicians in our focus groups were positive towards the use of a CDSS that would serve them with accurate and graded alerts focused on the important clinical issues. The CDSS has the potential to enhance their inter-professional collaboration. They understood well that the deficiencies in the system were due to a lack of correct information in the EHR system concerning patients’ medication and diagnoses. When implementing a CDSS, managers need to focus on common practices in the documenting of structured data within their organizations in order to prevent an undermining of trust in the system. The software developers should also focus on the ease of working with the documentation of structured data and on bringing relevant, timely and appropriate information to the screen for the clinician.

## Abbreviations

GFR: glomerular filtration rate
VAS: visual analogue scale
